# Prevalence and incidence of juvenile idiopathic arthritis in children in the republic of Bashkortostan: the epidemiological study

**DOI:** 10.1186/1546-0096-9-S1-P145

**Published:** 2011-09-14

**Authors:** VA Malievskiy

**Affiliations:** 1 Bashkir State Medical University, Ufa, Russia

## Background

Juvenile Idiopathic Arthritis (JIA) is the most common rheumatic disease of children. The results of reports vary considerably throughout the world. Therefore epidemiological research in field of paediatric rheumatology is very important.

## Aim

To investigate the prevalence and incidence of Juvenile Idiopathic Arthritis (JIA) in children in the Republic of Bashkortostan (Russia).

## Methods

Four towns and two rural districts of the Republic of Bashkortostan were chosen for the questionnaire. The total number of the childhood population was 50442 children under 17 years of age. Totally 43907 children or their parents were examined with the help of the screening questionnaire. The parents were questioned by medical nurses during consulting reception hours, or in kindergartens, or at school. The children aged between 11 and 17 years answered the question list on their own. The screening questionnaire included two questions:

1. Did you observe any pain in joints of your child?

2. Did you observe any swelling in joints of your child?

Hereinafter 368 children were included into the investigation, their parents having answered the question positively about the presence of joint swelling for the last year. 303 children (82.3 %) were examined in all.

Blood tests, joint radiography and medical examination by rheumatologist were held to all the children. The rest of 65 children did not come to the children’s polyclinic for the extra check-up and consultation.

## Results

After the all-round clinical, laboratory and instrumental investigation the diagnosis of JIA was defined in 35 children, seven of them being diagnosed for the first time during the year of 2003, which preceded the present investigation. According to the epidemiological study the prevalence of JIA was 83.9, and the incidence was 17.2 per 100,000 children under 16 years of age. The prevalence of JIA in boys was higher, than in girls (93.9 and 73.7 per 100,000 children, respectively, p < 0.001).

## Conclusion

The present investigation allowed to determine the prevalence and the incidence of JIA, which was 83.8 and 17.2 per 100.000 children under 16 years of age, respectively.

